# Sustainable Recovery of Preservative and Bioactive Compounds from Food Industry Bioresidues

**DOI:** 10.3390/antiox10111827

**Published:** 2021-11-18

**Authors:** Maria G. Leichtweis, M. Beatriz P. P. Oliveira, Isabel C. F. R. Ferreira, Carla Pereira, Lillian Barros

**Affiliations:** 1 Centro de Investigação de Montanha (CIMO), Instituto Politécnico de Bragança, Campus de Santa Apolónia, 5300-253 Bragança, Portugal; mariagabriela@ipb.pt (M.G.L.); lillian@ipb.pt (L.B.); 2 REQUIMTE—Science Chemical Department, Faculty of Pharmacy, University of Porto, Rua Jorge Viterbo Ferreira no. 228, 4050-313 Porto, Portugal; beatoliv@ff.up.pt

**Keywords:** bioresidues, value-added compounds, antioxidant molecules, green extraction methods, food applications

## Abstract

With the increasing demand for convenient and ready-to-eat foods, the use of antioxidants and preservative additives in foodstuff formulation is essential. In addition to their technological functions in food, bio-based additives confer beneficial properties for human health for having antioxidant capacity and acting as antimicrobial, antitumor, and anti-inflammatory agents, among others. The replacement of preservatives and other additives from synthetic origin, usually related to adverse effects on human health, faces some challenges such as availability and cost. An opportunity to obtain these compounds lies in the food industry itself, as a great variety of food waste has been identified as an excellent source of high value-added compounds. Large amounts of seeds, fibrous strands, peel, bagasse, among other parts of fruits and vegetables are lost or wasted during industrial processing, despite being rich sources of bioactive compounds. From a circular economy perspective, this work reviewed the main advances on the recovery of value-added compounds from food industry bioresidues for food application. Bioactive compounds, mainly phenolic compounds, have been largely obtained, mostly from seeds and peels, and have been successfully incorporated into foods. Additionally, alternative and eco-friendly extraction techniques, as ultrasound and microwave, have showed advantages in extracting antioxidant and preservatives compounds.

## 1. Introduction

According to a survey carried out between 2010 and 2011, it is estimated that about one-third of the food produced for human consumption in the world is lost or wasted, which represents ~1.3 billion tons per year [1]. The interest in estimating these values is not new; in 2007, Mahro & Timm [2] carried out a study about the possibilities of using food processing residues as a biomass resource and concluded that, despite the well-established state of food industry, reliable data on the amounts of the generated waste along the distinct processing stages were difficult to obtain.

More recently, this topic has been gaining interest and, in 2018, Corrado & Sala [3] published a review of existing studies on the generation of food waste on a global and European scale. Through this study, variations in food waste were estimated from 194 to 389 kg per person per year worldwide and from 158 to 298 kg per person per year in the EU. Among the reported works, the project FUSIONS (Food Use for Social Innovation by Optimising Waste Prevention Strategies) stands out, with the estimation of food waste generated at an European level [4]. This project was carried out between 2012 and 2016 through the 7th Framework Program of the European Community, and represented a milestone in the accounting of food waste, with the generation of a manual on food waste quantification [5].

The interest in estimating these values is in line with the concern for the reduction of food waste generated worldwide. In the EU, as summarized on the EUbusiness portal [6], the Waste Framework Directive (Directive 2006/12/EC) has been revised through the Directive 2008/98/EC, to encourage the reuse and the recycling of waste materials and to simplify existing legislation, establishing measures to protect the environment and human health. Another action was through the United Nations Sustainable Development Goal 12.3 [7], where member states have pledged to halve per capita global food waste, in retail and consumer levels, by reducing food waste along production and supply chains by 2030.

Achieving reduced food waste and losses has been the focus of several recent studies, which involves identifying the dynamics of bioresidues generation and developing mechanisms to avoid this generation, as also managing unavoidable losses through strategies such as reuse. In fact, a great variety of bioresidues has proven to be a valuable source for the recovery of high value-added compounds, mainly with antioxidant capacity [8,9,10,11,12,13,14,15].

In this context, this review aims to explore the potential of using these bioresidues and by-products, through the concept of a circular economy, for the extraction of bioactive compounds. It also addresses the application of green and environmentally-friendly extraction methods and the incorporation of the recovered compounds into foodstuff.

### Characterization

In addition to governmental interests in decreasing the volume of generated waste, views of characterization and ways of using these residues have also been gaining traction in the scientific circles [8,10,13,16]. The food waste data, discussed by Stenmarck et al. [4], were divided into the following sectors: primary production, processing, wholesale and logistics combined with retail and market, food services, and household; according to food waste quantification manual [5]. Through the results obtained, it is possible to verify that the biggest impact on the generation of waste comes from household and processing in the food manufacturing, in more detail 53% and 19%, respectively, considering food and inedible parts of food. The beginning of the food supply chain (primary production and processing) resulted in an estimated loss of approximately 26 million tons of food wasted in 2012 by the EU-28 [4]. Some cases of food waste are reported by the author Tristram Stuart in his famous book entitled “Waste: discovering the global waste scandal” [16], with the outstanding example of bread processing, which generates the waste of 4 slices from each loaf, the crust, and the first slice at either end, amounting to 13,000 slices of fresh bread wasted every day. However, the most impactful pictures are related to the waste generated at the harvest level, where thousands of fruits and vegetables are discarded for not meeting the sales standard, in terms of size and overall appearance.

FAO data [1] reports that in low-income countries, the generation of food waste is bigger during the initial and intermediate stages of the food supply chain. In industrialized countries, on the other hand, more than 40% of food losses occur at retail and consumer levels, despite also occurring at the beginning of the food supply chain in significant quantities. Analyzing data, it is also verified that, in Europe, more than 50% of the production of roots and tubers and ~46% of the production of fruits and vegetables are lost or wasted, considering the edible parts of food products produced for human consumption. The sub-Saharan Africa region has the lowest production volume of fruit and vegetable group, along with North America and Oceania, compared to other five groups of regions in the world (Europe, Industrialized Asia, North Africa, West and Central Asia, South and Southeast Asia, and Latin America), and ~20% of the amount is lost during industrial processing. 

In another study [17], it was found that, due to cumulative losses, the proportion of global agricultural dry biomass consumed as food is only 6% and 24.8% of the harvested biomass, i.e., only a small fraction of agricultural production is consumed as food. While losses during processing are also considerable (15–59% of processed crops), they widely vary between dry matter, energy, protein, and wet mass. 

This brief information reveals a huge lack in the use of food. The potential of waste in commercial exploitation and circular use will be discussed below, focusing on by-products generated at industrial levels. Table 1 summarizes recent works in terms of the characterization of the main components of nutritional and/or commercial value that can be recovered from food industry biowaste and explored for different purposes.

It is possible to highlight the fruit and vegetable group as the food raw materials that generate bioresidues with greater potential and interest for scientific study and commercial exploitation. This is because residues from fruit and vegetable processing are rich in organic matter, phytochemicals, and compounds with nutraceutical properties [31]. However, in general, diverse food residues are important sources of value-added compounds, with considerable levels of phenolic compounds, dietary fibers, polysaccharides, vitamins, carotenoids, pigments, oils, and others [32], justifying their recovery [33,34].

The works performed by Buratto et al. [14] and Cádiz-Gurrea et al. [27] are recent examples of that, as summarized in Table 1. In the first study, it was found that about 5–15% of the weight of açaí fruit is edible, being almost 80% of the weight of the total fruit composed of seeds and lost during processing. In the extract of these seeds, significant amounts of polyphenols and total flavonoids were identified, among other compounds, with high ORAC (oxygen radical absorbance capacity) value. In the second study, a value of ~60% for the generation of by-products from the processing of tropical fruits was accounted, where the bioresidues, composed mostly by peels and seeds, revealed to be rich in photochemical compounds.

## 2. Value-Added Compounds

The bioactive compounds (e.g., antioxidants, polyphenols, tocopherols, carotenoids, and vitamins) naturally present in food are important for human nutrition [35,36]. Found in cereals, fruits, vegetables, grains, and several other foods and its subproducts, these compounds are secondary plant metabolites [37,38] that can cause a significant impact in human and animal body functionality, with pharmacological or toxicological effects [39]. The primary metabolites are responsible for the growth and development of the plant, e.g., proteins, lipids, and carbohydrates [36,40]. On the other hand, and despite being considered as by-products of metabolic and biosynthetic pathways of those molecules, bioactive compounds exert significant functionality in plants. They exert, for example, protective support, acting as free radical scavengers; they are able to act as signalers, appealing to pollinating insects or seed dispersers; and can also provide defense, repelling insects, parasites or competing plants, among other diverse functions, through the many varieties of compounds [33,38].

When these bioactive compounds are incorporated into the diet, by food or medicinal bases, they can provide benefits to human health, developing positive effects on body functionality. Studies have linked the consumption of foods rich in bioactive compounds with a reduction in the risk of developing chronic diseases such as cancer, diabetes, obesity, and cardiovascular diseases [33,35]. The beneficial health effects exhibited by these compounds are due to their ability to modulate metabolic processes, such as antioxidant activity, enzyme inhibition or induction, inhibition of receptor activities, and induction and inhibition of gene expression [36]. In fact, the antioxidant activity is often highlighted among their functions [41,42,43], once they protect the body’s cells against oxidative damage, reducing the oxidative stress and preventing cancer, arteriosclerosis, and aging processes. These properties have been reported as highly suitable for preservative, fortifier, and stabilizer additives development [36].

Many different illnesses, such as cancer, cardiovascular and pulmonary diseases, neurological disorders, diabetes, arthritis, ageing process, and other neurological and endocrinological disorders, result from oxidative stress (OS). In turn, the OS is the result of an imbalance between the free radicals and the antioxidants on the metabolism, originated from environmental causes, inflammation processes, exposure to radiation, drugs, and others unfavorable conditions [39]. In this case, when the antioxidant defense enzymes are overwhelmed, the nutrients with antioxidant capacity, found in natural food, are important to help the body combating these free radicals. Thus, when significant amounts of bioactive compounds are part of the diet regularly, they are capable of exerting antioxidant activities [39,44].

There are several therapeutic properties and mechanisms of modulation of metabolic processes reported in the literature. In a recent review [45], the antiviral capacity of bioactive compounds focused on the COVID-19 management was highlighted. A two-way strategy to combat SARS Cov-2 infection using bioactive compounds as blockers has been reported: blocking S protein of the virus and blocking ACE2 receptor of the cell. In addition to nutraceutical properties, as previously measured, bioactive compounds are also known for their functional properties (antioxidant activity, solubility, absorption, coloring, stabilization, flavor, preserving, etc.). Natural pigments such as anthocyanins, carotenoids, and betalains are proposed as natural colorant additives in foods, being also linked to other benefits, such as antioxidant effects [46]. Carotenoids, e.g., lycopene, are also used in cosmetic products, due to its photo-protection capacity, protecting the skin from the sun [47]. In another study, Rodriguez-Garcia et al. [48] proposed the essential oil obtained from oregano as antimicrobial and antioxidant food additive. In the food industry, there is a growing interest in isolating these compounds from natural sources for further application in processed food, as alternatives to the use of synthetic additives (preservatives, nutritional additives, flavoring agents, coloring agents, texturizing agents, or miscellaneous additives). 

In general, numerous studies have been reporting that a great variety of food wastes are rich sources of bioactive compounds, with great potential for recovery and application in food, cosmetic, and pharmaceutical industries [38]. Vitamin D_2_, e.g., was recovered from the biological surplus remaining from the mushroom cultivation industry [49]; phenolic acids and flavonoids were obtained from tomato crop remains (pruning and end-of-cycle plant materials) [50], and organic acids, phenolic compounds, and high concentrations of anthocyanins were extracted from *Sicana odorifera* fruit epicarp [30]. The recovery of value-added compounds from bioresidues and their applicability in food will be further discussed in topic 4.

There are approximately more than 200,000 chemicals isolated and identified, considering primary and secondary metabolites, from higher plants worldwide [51]—such as proteins and amino acids, phenolic compounds, and other molecules with antioxidant activity, vitamins, dietary fiber and functional polysaccharides, minerals, fatty acids, enzymes, aromatic compounds, etc.—that can have added value by recovery from undervalued sources in agriculture and industry [52,53]. Note that the recovery of these compounds from natural sources is normally affected by some factors as the matrix properties of the plant materials and the extraction method conditions (e.g., solvent, temperature, pressure, and time) [54,55].

**Figure 1:**
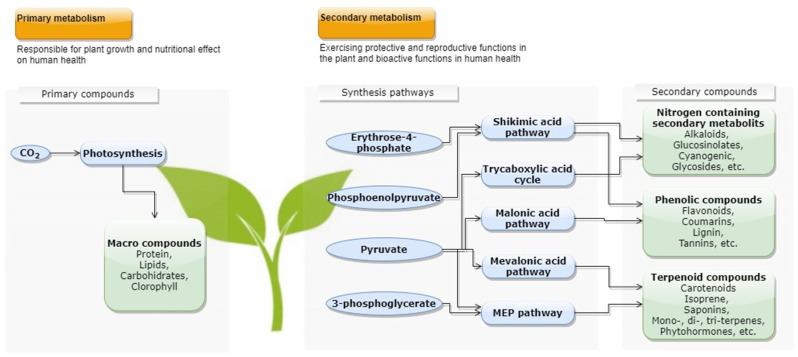
Simplified diagram of the synthesis pathways of secondary metabolites in plants and their groups of compounds. The different formation routes originate compounds with different characteristics and functionalities. Adapted from [37,38,40].

Bioactive compounds can be classified considering their solubility, polarity, and distribution in nature [39]. They are also commonly classified based on the biosynthetic route, the structural features, the basis of clinical function related to their pharmacological effect, and the botanical approach considering their families [38]. This classification results in a vast and heterogeneous number of classes of these compounds, e.g., phenolic compounds, carotenoids, tocopherols, alkaloids, and vitamins [37]. More details are presented through the diagram in Figure 1.

Phenolic compounds are one of the most widely found groups of secondary metabolites in plants [56]. They have a great structural diversity that, based on the number of constitutive carbon atoms conjugated with the structure of the basic phenolic skeleton, result in different subclasses: flavonoids, phenolic acids, stilbenes, lignans, tannins, and oxidized polyphenols [39,57]. These compounds are final products of the shikimate and acetate pathways, and their structure comprises aromatic hydroxylated compounds, having one or more aromatic rings with one or more hydroxyl groups. They can range from relatively simple phenolic molecules to highly polymerized compounds, most commonly being conjugated to mono- and polysaccharides, associated with one or more phenolic groups, and can also be linked to methyl esters and esters [51,57].

Among the more than 8000 known phenolic compounds, approximately 6000 constitute the group of flavonoids [51,56]. Flavonoid compounds are often stored in the plant linked to one or more sugars, being this form more stable than the free flavonoid [51]. These compounds generally have a yellow to red color and, when added to food products, they contribute to the color and flavor of food, in addition to being able to prevent oxidation of fats and protect vitamins and enzymes [43,51]. Anthocyanins, on the other hand, present red, blue, and purple colors, with a structure consisting of two aromatic rings linked by a three carbon heterocyclic ring that contains oxygen [46], while tannins, or tannic acids, exert antimicrobial action and are responsible for the astringency of many fruits, containing in its structure a large number of hydroxyl or other functional groups [51].

In turn, alkaloids and glycol sides are the categories, pointed by Vuong [38], which are currently attracting increasing attention for research regarding their recovery from industrial food bioresidues, once these natural nitrogen-containing organic compounds present great biological activities. For example, steroidal alkaloids were extracted from potato peel waste [58]; alkaloids were obtained from cocoa bean shell [59]; glycosides were extracted from pineapple waste [60], and quercetin glycosides from onion solid waste [61]. These groups of compounds, and others molecules, e.g., terpenoids and coumarins, were studied for their potential to protect against liver fibrosis [62]. The terpenoids are generally insoluble in water and based on five carbon units [37]. Carotenoids are an example of terpenoid compounds (tetraterpenes) widely explored from plant material.

## 3. Green Extraction Methods

The extraction of bioactive compounds from natural sources is an extensive topic of discussion. On the one hand, the seek to increase yield, optimize the parameters of extraction processes, and minimize the influences that affect the extraction and degradation of target compounds, in addition to reducing costs and process times. On the other hand, the challenge remains of obtaining the best results through methods that do not impact the environment nor the consumers’ health, reducing energy costs and using safe solvents.

The term green method is used to classify extraction methods that have certain advantages over conventional ones. While conventional methods generally require long extraction times to obtain greater performance and involve large amounts of solvent, which are sometimes toxic, alternative methods are more sustainable, using green solvents and more ecological techniques with high extraction yield and compounds preservation.

### 3.1. Extraction Solvents

Green solvents, in general, have non-toxic characteristics, are biodegradable, and obtained from renewable sources. In the databook of green solvents [63], it is possible to find more than 300 green solvents, with detailed information about usage considerations, physical properties, health and safety issues, potential substitutes, and for which products the solvent is recommended.

Among the green solvents, water is a great option with greenness characteristic as non-toxicity to health and the environment, safety, bioavailability, and price. In addition, it is possible to tune the properties of water by changing the temperature: at high temperatures, coupled to high pressure to keep the water in a liquid state (subcritical condition), the dielectric constant is decreased and the penetration of water into the sample matrix is favored, along with the decrease of the surface tension and viscosity, and the improvement of the analyte diffusion and mass-transfer kinetics [64]. Another well-known solvent is ethanol, a cheap and renewable solvent, produced by the fermentation of biological material, recognized as non-toxic, although flammable, but a final purification step is required in some processes. The equilibrium constant of this alcohol is strongly influenced by temperature, as the extractability of the material increases with temperature [65]. It has been presenting great selectivity to extract oil [65] and has been employed in many researches on the extraction of phenolic compounds, in hydroalcoholic solutions (see Table 2).

Recently, the large application of organic solvents such as benzene, toluene, xylene, methanol, and ethanol in many laboratories and industrial chemical processes has generated an environmental concern due to its high volatility, which contributes to global climatic changes, air pollution, and human health-related issues [66]. With this, new alternatives, such as supercritical fluids (SCFs), eutectic solvents (ESs), fluorous solvents, and ionic liquids (ILs), have been widely proposed [66,67,68].

The challenge of choosing the solvent involves considerations such as: being suitable for the method and efficient for the target compound and its matrix, economically viable, safe for health, and environmentally friendly. Some methods can help predict solvent performance through its physicochemical and thermodynamic properties. Mokashi et al. [69] used a mathematical model equation to estimate the extraction efficiency of various solvents for the recovery of pyruvic acid. The model relates Hansen Solubility Parameters (HSPs) with distribution coefficient, where HSPs is an equation of solubility parameters with different types of energy including polar, diffusivity, and hydrogen bonding interactions. In another work [67], a review of computational methods for screening green solvents is presented. The quantum chemistry (QC) methods based on continuum solvation models (CSM), used for solvent selection and design, and methods based on the Conductor-like Screening Model (COSMO) are discussed, which guides solvent selection from thermodynamic performance indicators.

### 3.2. Extraction Methods

#### 3.2.1. Principles

**Figure 2:**
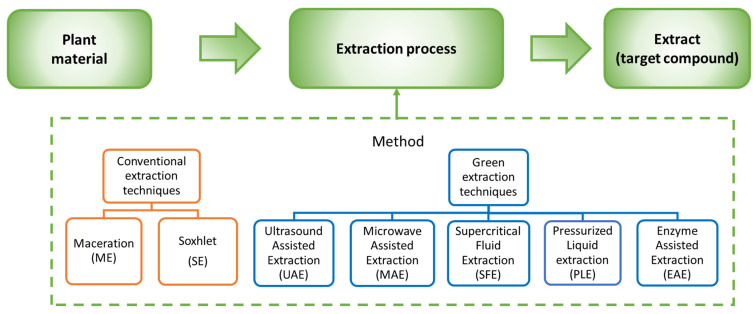
Simplified scheme of the extraction techniques employed to obtain target compounds from natural matrices.

The solid–liquid extraction process has been used for many decades, from home use to prepare tonics to a popular way of obtaining essential oils and bioactive compounds. The process consists of extracting the target compound by mixing the solid raw material with a solvent for a certain time. Thereafter, the solid–liquid phases are separated and the extract is purified. This technique is the principle of traditional extractions and many new enhanced extraction methods [70,71]. Figure 2 shows a simplified scheme of the main extraction methods employed in natural matrices. 

The dynamic of extraction is influenced by parameters such as the choice of the solvent, pH of the medium, solid–liquid ratio, process temperature, and contact area between the solid and the solvent. In turn, these variables affect the energy consumption, the quantity of solvent used and its recovery capacity, the extraction yield, and other factors, which are increasingly studied through the optimization of parameters and comparison between techniques for different target compounds and their matrix [13,70].

#### 3.2.2. Main Methods Explored in the Literature

Maceration (ME) consists of mixing the plant material with an appropriate solvent in a vessel and staying in contact for a certain (and usually long) time [72]. This process can be assisted by heating and/or stirring, e.g., thermostatic water bath, electro-magnetic stirring, and bain-marie with agitation. Generally, the solid matrix is ground to increase the surface area. It involves isolation and purification steps, usually by filtration and solvent evaporation [38]. 

Advantages and limitations: it is a simple and low-cost method, but requires long times of extraction and high quantities of solvent [73].

Soxhlet (SE) was originally developed for the extraction of lipids from a solid material, but it has also been adapted for bioactive compounds from various natural sources. In the Soxhlet equipment, the plant material is placed in a thimble of the apparatus and accoupled in a distillation flask containing the solvent, where the sample is washed by the solvent through intermittent reflux. When the solvent heats and condenses to an overflow level, a siphon system arrangement drains out the solution (solvent plus extracted solute) into the flask. There, the solution is heated until the solvent vaporizes and the process runs repeatedly for exhaustive extraction [74,75].

Advantages and limitations: it is a simple technique that demands less quantity of solvent, when compared to maceration, due to solvent recirculation. However, the extraction time is long and heat-labile compounds can be affected [73].

Ultrasound-assisted extraction (UAE) is based on the dispersion of sound waves in the liquid medium (solvent) that contains the sample to be extracted. Upon the waves reaching sufficient intensity of successive compression and distension in the medium, cavitation bubbles are formed. These bubbles, when collapsing, cause the rupture of cellular structures, facilitating the penetration of the solvent and increasing the mass transfer [76].

Advantages and limitations: it is an inexpensive and simple method, capable of improving extraction yield and faster kinetics [71], in addition to less consumption of energy, solvent, and extraction time. Nevertheless, the heat generated can affect heat-labile compounds and the reproducibility can be reduced by the aging of the instrument [76].

Microwave-assisted extraction (MAE) is a method that uses microwave radiation, i.e., non-ionizing electromagnetic waves (300 MHz to 300 GHz). Through this technique, an electric field is generated by the simultaneous effects of ionic conduction and dipolar rotation. The larger the dielectric constant of the solvent, the higher the heating and dipolar rotation. Through this mechanism, pressure is generated inside the plant cell and its consequent rupture, exposing the cell and then facilitating solvent penetration [77].

Advantages and limitations: it is a low-cost equipment, which requires reduced extraction time and quantity of solvent, with improved extraction yield. It also allows processing without using solvent. On the other hand, the heat generated can affect heat-labile compounds and it loses efficiency in scaling up [71,76].

Supercritical fluid extraction (SFE) has as principle the use of the solvent fluid in its supercritical state. For this, the temperature and pressure parameters of the process are controlled to achieve the conditions in which the fluid is between the gas and liquid states, presenting similar liquid density and gas viscosity [38]. Carbon dioxide (CO_2_) is an attractive solvent largely used in this method [78]. The supercritical solvent flows through the raw material, placed in an extractor vessel, transports the dissolved solute to the separator, and then can be regenerated and returned to the process [38].

Advantages and limitations: high selectivity for non-polar compounds, non-degradation of heat sensitive compounds, and non-residues of toxic solvents. However, it demands high costs and complex operation and training [76,78].

Pressurized liquid extraction (PLE) uses pressurized solvents at high temperatures and pressures, but without reaching the critical point values. Through this mechanism, it is possible to ensure rapid extraction rate, by decreasing the dielectric constant of the solvent [74].

Advantages and limitations: the use of pressure allows a faster extraction, with less solvents and higher yields, but, the high temperatures can damage thermolabile compounds [38].

Enzyme-assisted extraction (EAE) utilizes enzymes, as cellulase, xylanase, and pectinase, for example, capable to degrade the cell wall structure, facilitating the extraction of many bioactive compounds, to which the access is often hindered because they are linked to the constituents of the cell wall [79].

Advantages and limitations: it presents high selectivity and improves yield, however, the enzymes are expensive and demand rigorous control of medium pH and temperature for optimal enzyme action [76].

### 3.3. Extraction Parameters

Despite the summarized contextualization of the principles of the most common extraction methods, there is a great deal of processes and combinations of these in the literature. Table 2 presents some recent extraction studies and the used parameters, as well as the conditions optimized and/or highlighted by the authors.

There are several factors that can influence the success and yield of the extraction. Parameters as solvent type, liquid–solid ratio (LSR), particle size, temperature, time of exposure, power, and pressure, are commonly studied. To outline the best conditions of the extraction procedure for different plant materials and target compounds, optimization studies are very important. For this, the most relevant independent parameters are evaluated, in a predetermined range of values, combined (or not) with the comparison of different extraction methods.

For instance, anthocyanin-rich extracts were obtained in optimization and comparison studies of heat- and ultrasound-assisted extraction techniques, using different fruits as sources: *Arbutus unedo* L. fruits [80], *Prunus spinosa* L. fruit epicarp [13], and *Ficus carica* L. fruit peel [81]. These works followed the same evaluation structure, however, the alternative UAE method proved to be more efficient for *F. carica* and *P. spinosa*, while for *A. unedo* the conventional method (heat-assisted extraction) was the most effective in the evaluated responses. These results demonstrate the influence of the matrix on the method’s performance. In another study [74], the conventional method (Soxhlet) also stood out when compared to the emerging technology pressurized liquid extraction (PLE), for the extraction of phenolic compounds and mannitol from olive leaves. However, although the Soxhlet extraction revealed a better performance in most of the analyzed variables, the authors highlighted shorter extraction times (5 min versus 4 h), and lower solvents consumption and energy costs as advantages achieved by PLE method, which is important to be considered in industrial scale application. 

Reduction of extraction time and energy consumption was also reported by Jesus et al. [82], by using the microwave-assisted extraction method in comparison to the heat-assisted one. In addition to these advantages, through the alternative method, the authors achieved higher yields and concentrations of ellagic acid. The conventional maceration technique was compared to the heat and ultrasound assisted extraction processes to obtain polyphenols from *Thymus serpyllum* L. herb [83]. In this work, the UAE was the most efficient, followed by the HAE (heat-assisted extraction) and, last, the ME. The authors discussed the influence of the process time and, in the preliminary screening, the high time of exposure caused a slight decline in the polyphenol content. On the other hand, in the results obtained in the optimization study (running in low temperatures), time did not significantly affect the extraction, with the relevant factors being the particle size, solid-to-solvent ratio, solvent type, and extraction procedure. 

Other studies reported enzymes being used as pretreatment for supercritical carbon dioxide extraction of lycopene from tomato [84]. The use of plant cell wall glycosidases led to a significant increase in the concentrations of lycopene and total lipids in the matrices, when compared to the control. On the other hand, Jiang et al. [68] defended the use of deep eutectic solvents (DESs) as an alternative to organic solvents for more efficient and green extraction. DESs are a mixture of two or more hydrogen bond acceptor and donor, bound together by strong intermolecular interactions, which are being proved as new high-efficiency extraction solvents. In this study, the authors optimized an efficient DESs extraction method to different types of bioactive alkaloids. Moreover, Babova et al. [85] combined the supercritical (SC) and subcritical (SubC) CO_2_ extraction methods in a multistage process to improve the extraction of antioxidant compounds (anthocyanins, other flavonoids, other phenolics, and proanthocyanidins) from bilberry (*Vaccinium myrtillus* L.). This methodology proved to be efficient showing great performance of selectivity through the stepwise extraction procedure. Other high-pressure methods (SFE and PLE) were compared to low-pressure methods (SE and UAE) to obtain an ethanolic extract with high antioxidant potential from black pepper [86]. The authors pointed out that each extraction procedure presented particular characteristics, but, in general, SE and UAE extracts presented higher yields when compared to SFE method; PLE has shown good results to the extraction of bioactive compounds; and SFE showed effectiveness for the recovery of extracts rich in piperine, providing a high value-added and solvent-free extract.

**Table 2:** Optimized green methods for the extraction of natural compounds.

Method	Source	Compound	Solvent	Extraction Conditions	Reference
Maceration extraction (ME)	*Eucalyptus globulus* L. leaves	Phenolic compounds (mostly flavonoids)	Ethanol	LSR 20 L/Kg, 2 Hz, 50 °C, 225 min, 56% ethanol	[87]
	*Arbutus unedo* L. fruits	Anthocyanins	Ethanol (pH 4, 0.05% of hydrochloric acid)	LSR 5–40 L/Kg, 8.33 Hz, 5 min, 90 °C, 80% ethanol	[80]
Microwave-assisted extraction (MAE)	Lamiaceae (*Origanum glandulosum* Desf.)	Phenolic compounds (mostly gallocatechin)	Ethanol	LSR 15 L/Kg, 850 W/2455 MHz, 42 °C, 2 min, 0% ethanol	[77]
	Lamiaceae (*Thymus fontanesii* Boiss. et Reut.)	Phenolic compounds (mostly rosmarinic acid)	Ethanol	LSR 15 L/Kg, 850 W/2.455 × 10^9^ Hz, 150 °C, 9.5 min, 50% ethanol	
	*Coriolus versicolor* (L. ex Fr.) Quél. mushroom	Phenolic compounds	Ethanol	LSR 10 L/Kg, 125 W, 3.8 min, 40% ethanol	[88]
	Vine pruning residue	Phenolic compounds (mostly ellagic acid and apigenin)	Ethanol	LSR 40 L/Kg, 120 °C, 5 min, 60% ethanol	[82]
	*Morus nigra* L. fruits	Phenolic compounds (mostly anthocyanins)	Ethanol	LSR 50 L/Kg, 500 W, 35 °C, 10 min, 35% ethanol	[89]
	*Arbutus unedo* L. fruits	Flavonoids	Ethanol	LSR 20 L/Kg, 400 W, 120 °C, 1.5 min, 0% ethanol	[72]
Ultrasound assisted extraction (UAE)	Goji berry fruit	Carbohydrates and phenolic compounds	Water	LSR 28 L/Kg, 283 W, 64.29 °C, 39.7 min	[90]
	*Thymus serpyllum* L. herb	Phenolic compounds	Ethanol	LSR 30 Kg/L, 25 °C, 15 min, 50% ethanol (750 W output with a 2 × 10^4^ Hz converter)	[83]
	*Prunus spinosa* L. fruit epicarp	Anthocyanins	Ethanol (pH 3, citric acid)	LSR 20 L/Kg, 400 W, 5 min, 47.98% ethanol	[13]
	*Ficus carica* L. peel	Anthocyanins	Ethanol	LSR 6.66 L/Kg, 310 W, 21 min, 100% ethanol	[81]
	*Tarchonanthus camphoratus* L. leaves	Parthenolide	Ethanol	LSR 20.4 L/Kg, 38.8 °C, 50 min, 100% ethanol	[91]
	Agro-industrial acerola (*Malpighia emarginata* DC) residue	Anthocyanins, other flavonoids, other phenolic compounds, carotenoids, and ascorbic acid	Ethanol (pH 2, hydrochloric acid 2 mol/L)	LSR 8.66 L/Kg, 50 kHz and 250 VA, 30 °C, 49.30 min, 46.49% ethanol	[92]
Subcritical fluid extraction (SubFE)	Black mulberry, wall germander, wild geranium, and comfrey	Phenolic compounds (mostly gallic acid)	Water	LSR 40 L/Kg, 160 °C, 1 × 10^6^ Pa, 3 Hz, 30 min	[93]
	Chestnut shells	Phenolic antioxidants (mostly caffeoylquinic acid isomers)	Water	LSR 10 L/Kg, 220 °C, 4 × 10^6^ Pa, 30 min	[94]
Supercritical fluid extraction (SFE)	Canola seeds	Tocopherol-rich oil	Carbon dioxide (CO_2_)	70 °C, 8 × 10^6^ Pa, 30 min, 1.67 × 10^−5^ L/s	[78]
	Raspberry seeds	Oil	Carbon dioxide (CO_2_)	40 °C, 3.5 × 10^7^ Pa, 240 min, 1.11 × 10^−4^ Kg/s	[95]
	Apple seeds	Fatty acids rich oil (mostly linoleic acid)	Carbon dioxide (CO_2_)	40 °C, 4 × 10^6^ Pa, 140 min, 2.78 × 10^−4^ L/s	[96]
Pressurized liquid extraction (PLE)	Olive leaves	Phenolic compounds and mannitol	Ethanol	LSR 3 × 10^−3^ Kg dw sample into 2.2 × 10^−2^ L stainless steel cells, 190 °C, 5 min, 60% ethanol	[74]
	Pomegranate peels	Condensed tannins, anthocyanins, other flavonoids, other phenolic compounds	Ethanol	LSR 60 L/Kg, 4.92 × 10^8^ Pa, 20–38 °C, 30 min, 37% ethanol	[97]
	Truffles *Tuber melanosporum* Vittad.	Sterols and β-glucans	Ethanol and water	5 × 10^−4^ Kg, 1.67 × 10^7^ Pa, 180 °C, 30 min, 100% ethanol or water	[98]

LSR: liquid–solid ratio.

## 4. Value-Added Compounds in the Development of Innovative Food Products

The recovery and valorization of compounds through green methodologies and from bioresidues, adding value to what would be wasted, corroborates the principles of a circular economy [99]. The circular economy is the concept related to the reduction, reuse, and recycling of food losses and wastes along the food supply chain [100]. The use of bioresidues to obtain value-added compounds is a management strategy for waste and food loss, which can contribute to reducing its environmental impacts, minimizing the use of virgin materials, in addition to promoting opportunities for savings, as innovation of products and methods, competitiveness and productivity [100].

Innovation in the food industry through the use of biocompounds not only adds to the circular economy sustainability concept but is also an opportunity to attend consumers’ expectations. In fact, consumers’ concern for safer and healthier foods is increasing, and food industry is under pressure to offer healthy, convenient, and ready-to-eat foods, able to meet daily nutritional needs, provide pleasure and satiety, and attend to consumers’ expectations and safety issues [101,102]. In this scenario, the replacement of synthetic compounds, generally associated with toxicity and allergenic problems, with healthy natural alternatives is increasingly evident [101]. Alongside, the enrichment of products by using compounds with nutritional value is also a growing tendency. The use of agro-industrial residues, rich in bioactive compounds, has been the focus of studies that propose the use of these by-products in the formulation of functional foods [102]. As synthetized in Table 3, value-added compounds were recovered from industrial processes wastewater, from commercially unexplored fruits, and industrial processing by-products, and their applicability was verified. Below, some relevant works available in the literature on the valorization of these compounds and practical applications in foodstuff are discussed.

For example, potato peel wastes were valorized as a source of protein and dietary fiber through their addition to cake [109]. The protein and soluble and insoluble fiber contents of the potato peel powders were about 15%, 19%, and 10%, respectively. The cakes enriched with 10% of potato peel flour achieved a percentage of protein improvement of ~17%. Regarding dietary fiber, the soluble fiber content increased from 3.3% in control cake to almost 5% in enriched cakes, and the insoluble fiber content significantly increased from 15.9% (control) to ~22% for cakes with potato peel flour. In addition to improving the nutritional value, the authors reported technological effects: the incorporation of potato peel powder at 5% increased the dough strength and elasticity-to-extensibility ratio.

Grape seeds and apple peels were also valorized as sources of natural antioxidants, especially phenolic compounds, through the fortification of yogurts with these bioresidues powders [110]. In this line, the authors also optimized the extraction conditions, using green solvents, to obtain extracts with high phenolic compounds content from these by-products. In another study, Chen et al. [111] discussed the applications of grape seed extract in food industry as preservative. They proposed the use of the extract as raw material to develop healthy foods as it improves the nutritional value and promotes benefits such as enhancing the body immunity, prevent hyperlipidemia, hypertension, and diabetes; as natural antioxidant and preservative in food, due to its antioxidant and antimicrobial activity; as food film/coating in food packaging, to improve certain functional properties; and as substitute of nitrite and nitrate in meat products, and sulfur dioxide (SO_2_) and animal protein in wine making.

Moreover, the ethanolic extracts of apple peels were fractionated and their use for inhibition of fish oil oxidation was studied using the thiobarbituric acid reactive substances (TBARS) assay [112]. The crude and fractionated extracts presented inhibitory effect on fish oil oxidation, where the greatest antioxidant capacity was verified with the fractions containing quercetin glycosides and epicatechin in combination with other polyphenols, such as phloridzin and cyanidin-3-galactoside. The apple peel was also successfully used as prebiotic in yoghurt [113]. Through this study, a probiotic yoghurt fortified with apple peel polyphenol extract was obtained, which can act as natural high-quality antioxidant and bioactive compound.

In another study [114], a green extraction method was used to obtain phenolic compound-rich extracts from olive leaves. The extracts were obtained by solvent-free MAE and presented high antioxidant activity, so they were proposed as having a great potential as functional ingredients for food packaging. In fact, the developed biodegradable films based on carrageenan containing olive leaf extract showed good barrier and mechanical properties, and the total phenolic compounds and antioxidant activity of the films significantly increased with increased concentrations of the olive leaf extract.

Promising antioxidant extracts were also obtained from the peel of eggplant. Horincar et al. [115] used the green method of UAE to obtain a methanolic extract of this by-product. Six anthocyanins were identified in the extract: delphinidin-3-rutinoside, delphinidin-3-glucoside, cyanidin-3-rutinoside, delphinidin-3-rutinoside-5-glucoside, malvidin-3-rutinoside-5-glucoside, and petunidin-3-rutinoside. In a subsequent study [116], the extract was microencapsulated with whey protein and acacia gum, resulting in a purple colored powder. The addition of the eggplant peel powder in a pastry cream allowed a significant increase of total phenolic content and antioxidant activity, which were rather stable over 72 h of storage under refrigeration conditions. The ethanolic extract of eggplant peel was, then, proposed as supplement in beer [117], with the supplemented beer presenting high functional potential and good sensory characteristics, being stable without the incorporation of artificial preservatives.

Anthocyanin-rich extracts from blackthorn epicarp [13] and fig peel [81], obtained by a green optimized extraction method (previously discussed in Section 3.3), were proposed as alternative natural colorants. The extracts were incorporated in confectionery products, more specifically “beijinhos” (a typical Brazilian pastry) and doughnut icing. The obtained purple colorant extracts conferred attractive color to the products, improved the texture properties, and significantly increased the antioxidant and antimicrobial activities. In fact, anthocyanins are widely found in many fruits. As reviewed by Albuquerque et al. [118], fruits and their bioresidues are an excellent source of natural compounds, including a wide range of coloring, in addition to bioactive properties and with great potential to be implemented in the food industry as alternative to the use of synthetic additives. Moreover, the bioresidues from food industry of *Morus nigra* L. and *Rubus fruticosus* L., for not presenting adequate size or properties to be marketed, were also studied as sources of anthocyanins [119]. The juices from these fruits were used in the preparation of solid colorants using the spray-drying technique, which resulted in colorants with a great and stable coloring capacity over time and safe for application in the food industry. In another study [120], an anthocyanin-rich extract was obtained from purple and red potatoes and evaluated as natural colorant in a soft drink formulation in comparison with the commercial colorant E163. The extracts showed suitable profiles in the sensory and shelf-life assessments, with high color stability during a 30-day shelf-life. Despite their multiple health benefits, some of these fruits are not used for consumption for not presenting the suitable size or properties to be included in the market, constituting a food industry residue. 

Furthermore, as alternative for the use of synthetic additives in food industry, sage (*Salvia officinalis* L.) and basil (*Ocimum basilicum* L.) were exploited for their preservative purposes [121]. For that, extracts were obtained and incorporated into yogurt. The results were very satisfactory, with the extracts presenting antioxidant and antimicrobial activity, without changing the physicochemical and nutritional characteristics of the yogurts and the growth of lactic acid bacteria.

However, there is a wide range of sources to be explored and valued. In the literature, several biowastes were characterized and identified as having great potential for the recovery of value-added compounds, which could be applied in the food industry. Grape (*Vitis vinifera* L. var. Albariño) and mulberry (*Morus nigra* L.) seed pomace was characterized and the first presented high contents of organic acids and phenolic compounds, mainly catechins, while the mulberry seeds revealed to be rich in tocopherols and ellagic acid derivatives. The extracts containing these compounds showed antioxidant and antimicrobial activity and no cytotoxicity on PLP2 cells (a primary culture of porcine liver non-tumor cells), being their use proposed as natural preservative in the food industry. The epicarp of the eggplant fruit (*Solanum melongena* L.) was highlighted by the authors [122] as a potential natural source of coloring compounds for food application, once it is rich in anthocyanins. Cereal by-products from the flour milling industry, more specifically wheat germ, maize bran–germ mixture, rye bran, and wheat bran, were reported [123] as underexploited alternative sources of nutrients and bioactive compound, such as protein and vitamin E.

## 5. Conclusions

Bioresidues from food industry have a great potential for the recovery of many value-added compounds. In the inedible parts as seeds, peel, and bagasse, as well as edible but rejected raw materials, many nutritional and bioactive compounds with high antioxidant capacity have been identified. The valorization of by-products and bioresidues, and the recovery of these compounds through green and environmentally friendly methods goes towards a circular economy. In the literature, several reports of the successful application of compounds obtained from food waste can be increasingly found. However, the transition to a circular food system with an efficient use of resources and food distribution is still a long challenge. There is a huge quantity of residues and unexploited plants to be characterized and valorized, and extraction processes to be optimized, leading to energy, solvent, and time reduction, among other relevant parameters. Studies on the stability of the recovered compounds, their stabilization through innovative techniques, and application in different matrices, as well as the scale-up process from laboratory to industrial level are also of great importance and increasingly needed. In general, this review aimed to contribute with new perspectives on underexploited and wasted biomaterials, aiming at the sustainability in industrial processes with the consequent increase in the use of natural compounds compared to artificial ones, meeting the emerging expectations of consumers, and promoting a circular food economy.

## Figures and Tables

**Table 1:** Biocompounds found in bioresidues from food industry.

Industrialization Process	By-Product(s)/ Bioresidue(s)	Biocompounds	References
Processed potatoes	Peel	Polyphenols, antioxidants, proteins, polysaccharides, vitamins, and minerals	[8,9]
Depulping of juçara fruit	Pomace and seeds—about 74%	Antioxidants compounds and unconventional starch	[10]
Pumpkin processing	Peel, seeds, and the flesh between seeds	Phenolic compounds and carotenes	[11]
Kiwi fruit processing	Skin and bagasse	Dietary fiber and bioactive compounds with antioxidant activity	[12]
Blackthorn fruit processing	Epicarp	Anthocyanins	[13]
Açaí fruit processing	Seeds, slurry, and pulp residue—about 80 to 95%	Antioxidant compounds, polyphenols and flavonoids, cellulose, hemicellulose, and lignin	[14]
Passion fruit processing	Rinds and bagasse—about 40 to 60%	Tocopherols and tocotrienols, fatty acids, carotenoid, and phenolic compounds (mostly piceatannol)	[18]
Wine production	Bagasse (grape skins and seeds), stalks, and sludge—about 30%	Organic matter, phytochemicals, and compounds with nutraceutical properties	[15]
Cashew nut processing	Shell liquid, testa, cashew apple, and cashew apple bagasse	Bioactive compounds, polymers, and potent lignocellulosic material	[19]
Tamarind processing	Peel, fiber, and seeds—about 50 to 70%	Phytochemicals, mainly polyphenols, fatty acids, and polysaccharides	[20]
Sugar cane industry	Sugarcane bagasse	Cellulose, hemicellulose, and lignin	[21]
Cereal grinding	Cereal bran	Minerals, phenolic acids, amino acids, and vitamins	[22]
Beer production	Spent grain	Oligosaccharides, for example arabino-xylooligosaccharides (prebiotic nutraceutical)	[23,24]
Tomato processing	Peels or mixture of peels, seeds, and a small amount of pulp	Carotenoids, mainly lycopene	[25]
Vanilla extract processing	Bagasse of the pod	Water- and ethanol-insoluble aromatic compounds	[26]
Tropical fruit processing	Seeds, peels, and leaves—up to 60%	Phenolic compounds, carotenoids, proteins, vitamins, or dietary fibers	[27]
Sardine processing	Waste from canning facility—about 20 to 75%	Proteins, peptides, amino acids, lipids (omega-3 polyunsaturated fatty acids, PUFAs), enzymes (pepsin, trypsin), vitamins (A, D, E) and biopolymer	[28]
Jabuticaba processing	Epicarp	Antioxidant compounds, tocopherols, anthocyanins, and ellagitannins	[29]
Melon croá prossessing	Epicarp	Anthocyanins and tocopherols	[30]

PUFA: polyunsaturated fatty acids.

**Table 3:** Value-added compounds recovery from food waste and its applicability.

Compound(s) of Interest	Source(s)	Benefit for Health	Applicability	Reference
Vitamin D_2_	Surplus mushrooms	Antitumoral	Food industry	[49]
Anthocyanins	Fig peel and blackthorn fruit epicarp	Antioxidant and antimicrobial activities	Natural purple colorant in pastry products	[41]
Dietary fiber	Pumpkin seeds and rinds	Nutritional value	High fiber bakery product	[103]
Phenolic compounds	Peel of camu-camu fruit	Antimicrobial potential	Yogurt fortification	[104]
Anthocyanins	Strawberry tree fruit	Antioxidant and antifungal activities	Natural colorant in wafers	[42]
Phenols	Olive mill wastewater	UVA and UVB filter potential	UV booster in cosmetics	[105]
Sugar	Coffee silverskin and spent coffee grounds	N.A. ^¹^	Ethanol production by fermentation	[106]
Phenolic acids, hydrolysable tannins, flavonoids, and anthocyanins	Pomegranate epicarp	Antioxidant and antibacterial activities	Natural colorant and antioxidant in pastry products	[43]
Phenolic and carotenoid compounds	Pumpkin peel	Antioxidant activity	Retard canola oil oxidation	[107]
Anthocyanins	Jabuticaba epicarp	Antioxidant, antimicrobial, antitumor and anti-inflammatory activities	Natural colorant in macarons	[108]

^¹^ Not Applicable.
